# Supplementary material for: Scanning electrochemical microscopy screening of CO2 electroreduction activities and product selectivities of catalyst arrays
Source: Commun Chem. 2020 Nov 6;3:155. doi: 10.1038/s42004-020-00399-6 (PMC9814292; doi:10.1038/s42004-020-00399-6)
Supplement: Supplementary file 1 — Supplementary Information [file 42004_2020_399_MOESM1_ESM.docx]

**Supplementary Information for:**

**Scanning Electrochemical Microscopy Screening of CO_2_ Electroreduction Activities and Product Selectivities of Catalyst Arrays**

Francis D. Mayer^1^, Pooya Hosseini-Benhangi^2,3^, Carlos M. Sánchez-Sánchez^4^, Ed Asselin^5^, Előd L. Gyenge^1,*^

^1^ Dept. of Chemical and Biological Engineering, Clean Energy Research Centre, The University of British Columbia, 2360 East Mall, Vancouver, Canada, V6T 1Z4

^2^ Dept. of Materials Engineering, The University of British Columbia, 6350 Stores Road, Vancouver, BC, Canada V6T 1Z4

^3^ Agora Energy Technologies Ltd., 3800 Wesbrook Mall, Vancouver, BC, Canada V6S 2L9

^4^ Sorbonne Université, CNRS, Laboratoire Interfaces et Systèmes Electrochimiques, LISE, 75005 Paris, France

^5^ Dept. of Materials Engineering, Canada Research Chair in Aqueous Processing of Metals, The University of British Columbia, 6350 Stores Road, Vancouver, Canada, V6T 1Z4

^*^Corresponding Author: elod.gyenge@ubc.ca





Supplementary Figure 1: Morphology created by electroreduction of mirror-polished native Sn/SnO_x_ substrates at electroreduction potentials of: a) −1 V_Ag/AgCl_ b) −1.5 V_Ag/AgCl_ c) −2 V_Ag/AgCl_ d) −2.5 V_Ag/AgCl_ e) −3.5 V_Ag/AgCl_ f) −4 V_Ag/AgCl_.





Supplementary Figure 2: Morphology created by electroreduction of different types of tin oxide at a potential of −3 V_Ag/AgCl_ for 30 min. a) Electrochemically formed tin oxide: −0.1 V_Ag/AgCl_ for 5 min. after pre-electroreduction at -1.25V_Ag/AgCl_ b) Chemically formed tin oxide: 1 M HNO_3_ for 1 min.





Supplementary Figure 3: Proposed combined reaction mechanisms for formate oxidation reaction and CO oxidation reaction on Pt. **^1–3^**





Supplementary Figure 4: Reaction mechanism for hydrogen oxidation reaction on Pt.**^4,5^**


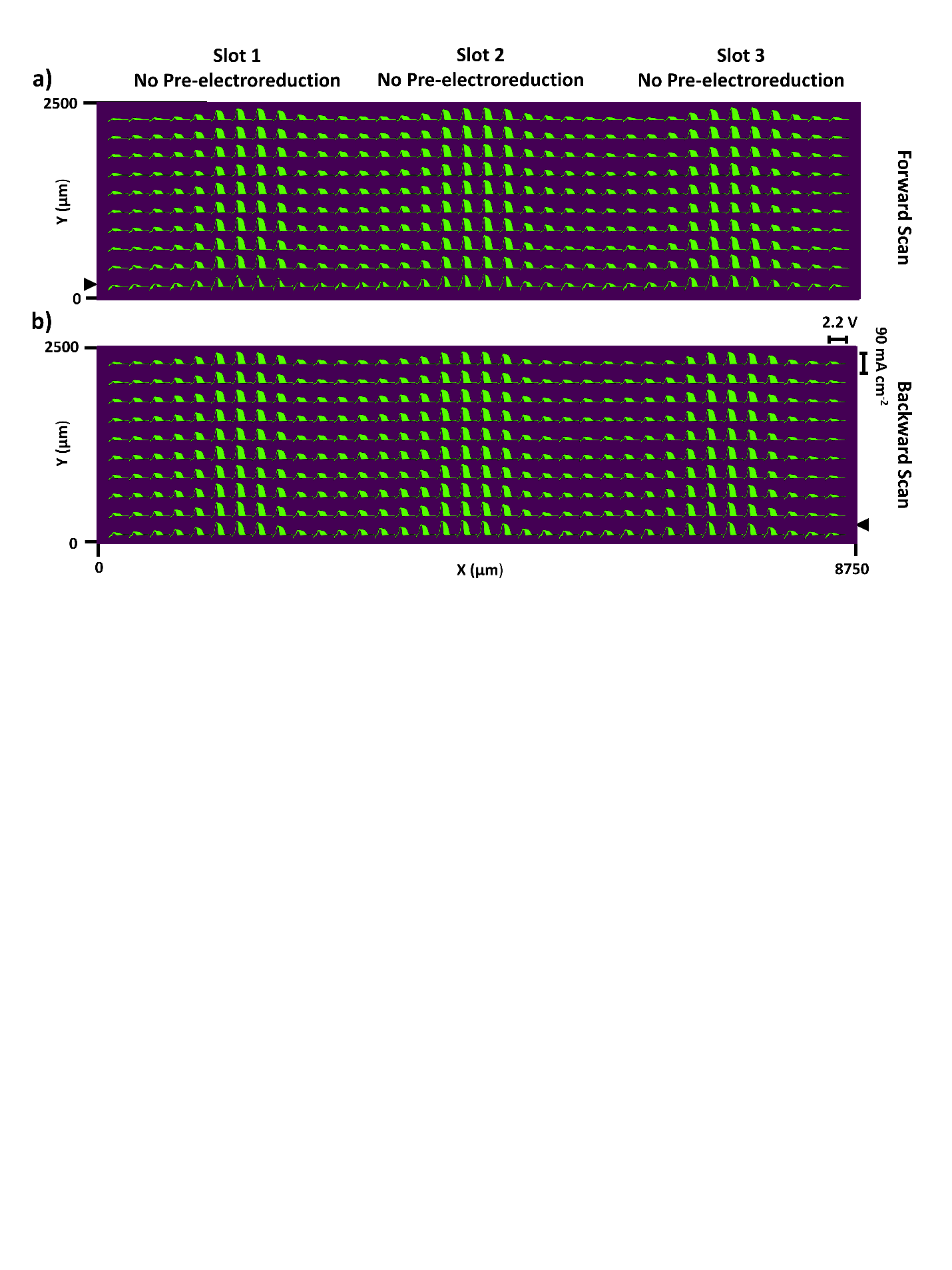


Supplementary Figure 5: CV-SECM scans after prolonged (2 hrs) exposure of an array composed of three identical unreduced (native) Sn/SnO_x_ catalysts under the approach curve conditions (0.1 M KCl, pH 8.75, air saturated). During CV-SECM scans the array of three identical unreduced (native) Sn/SnO_x_ catalysts was at −1.5 V constant substrate potential. a) Forward CV-SECM scan, b) Backward CV-SECM scan. CV scan rate 1 V s^−1^. CV range = 1.2 to −1.0 V_Ag/AgCl_ . Tip-substrate distance: 100 μm, tip scan rate: 100 μm s^−1^. Electrolyte: 0.1 M KHCO_3_ saturated with CO_2_ at atmospheric pressure. 293 K. Pixel size: 250x250 µm.


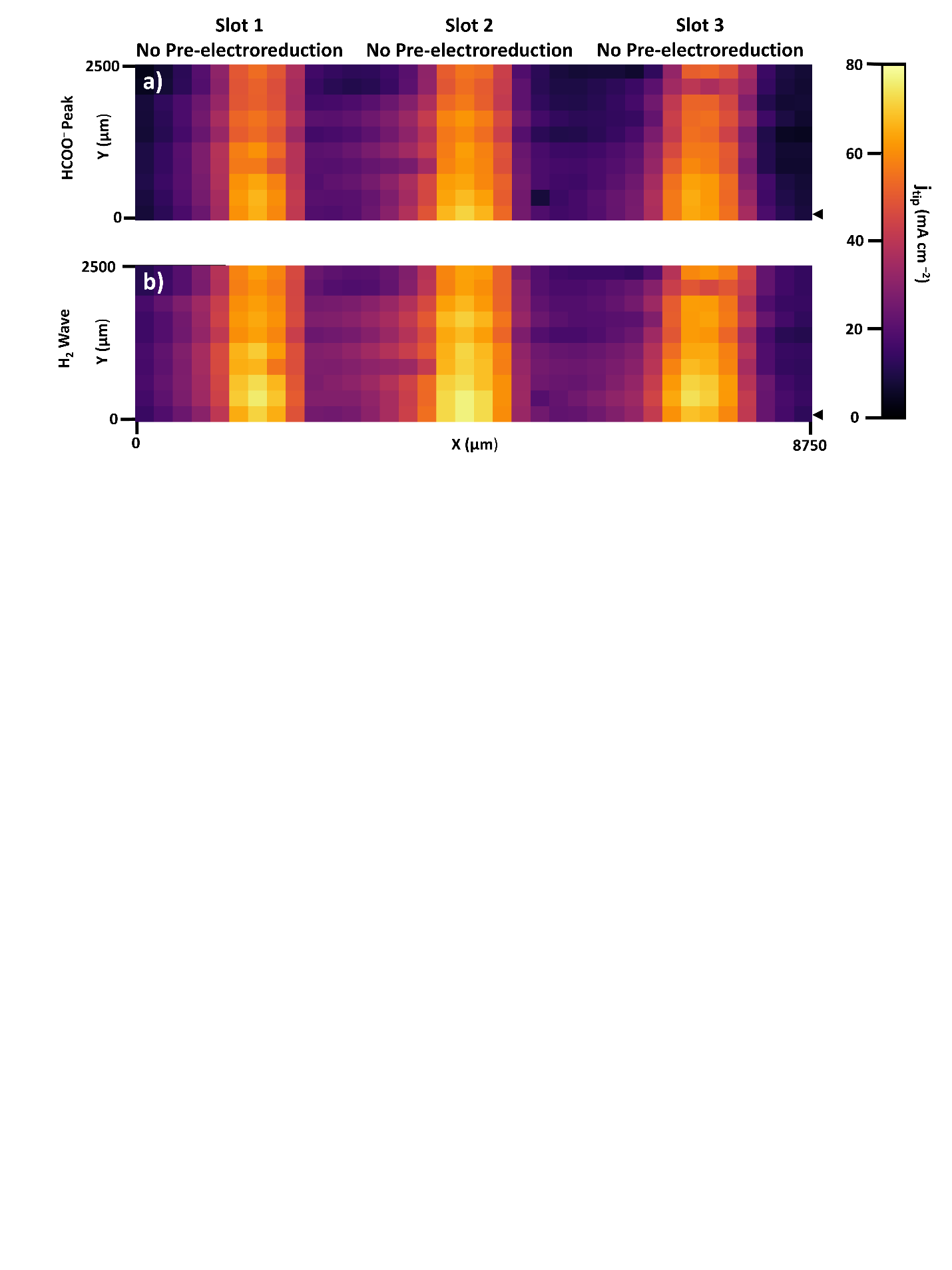


Supplementary Figure 6: Current densities extracted from the CVs collected in the backward scan of the CV-SECM performed on three identical unreduced (native) Sn/SnO_x_ catalysts according to the conditions described in Fig. S5B. a) HCOO^−^ peak, b) H_2_ wave. CV scan rate 1 V s^−1^. CV range = 1.2 to −1.0 V_Ag/AgCl_ . Tip-substrate distance: 100 μm. Tip scan rate: 100 μm s^-1^. Electrolyte: 0.1 M KHCO_3_ saturated with CO_2_ at atmospheric pressure. 293 K. Pixel size: 250x250 µm. The black arrow indicates the starting position of the tip.


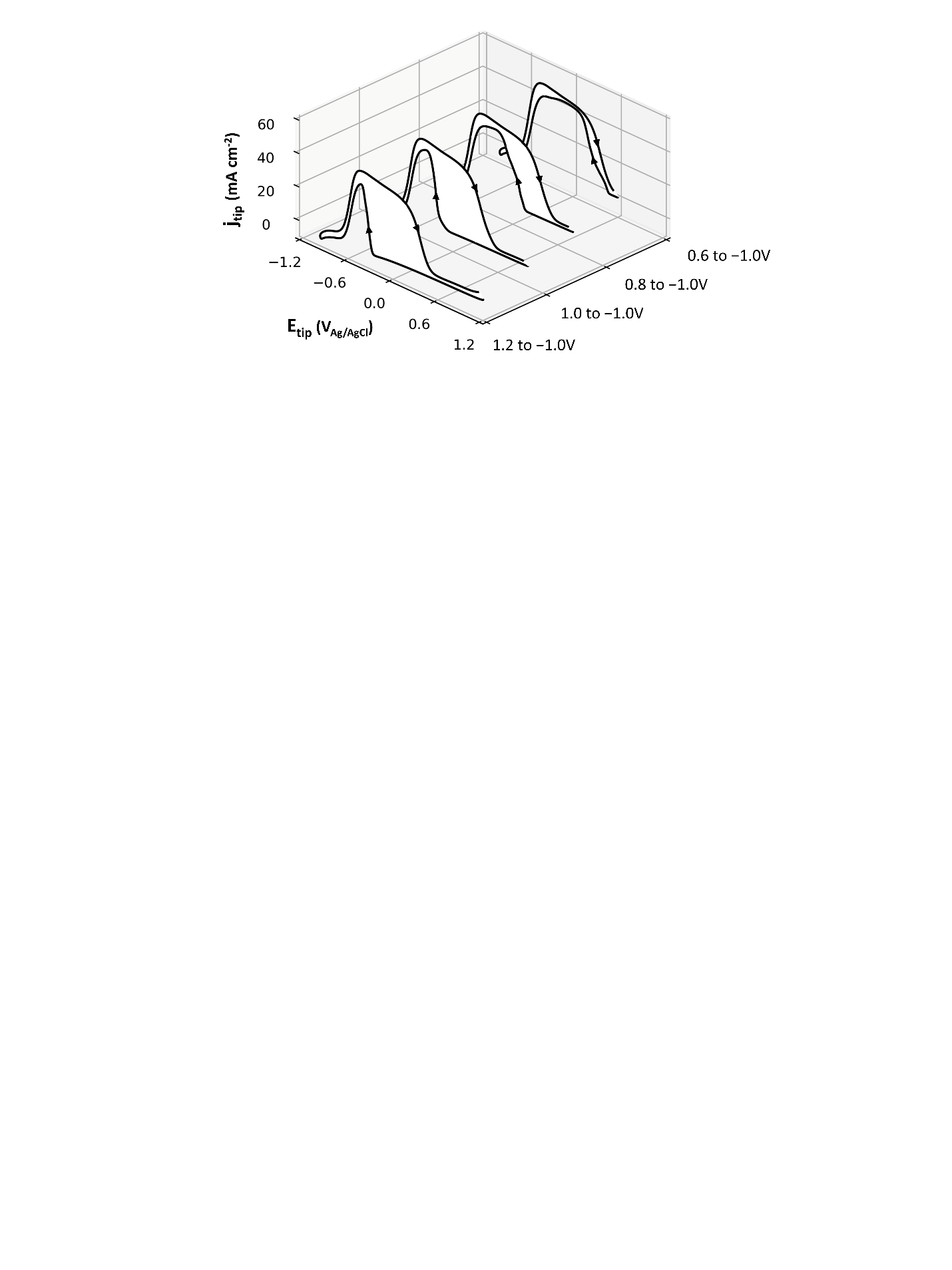


Supplementary Figure 7: Effect of Pt UME tip potential sweep range for *in-situ* detection of formate generated on the native Sn/SnO_x_ (i.e., no pre-electroreduction) catalyst substrate: The 50^th^ cycles are shown. Note: Before data acquisition, the tip was held at the starting potential for 10 s to simulate SECM scanning conditions. Electrolyte: CO_2_ saturated (at atmospheric pressure) 0.1 M KHCO_3_. 293 K. Scan rate: 1 V s^−1^. The tip-substrate distance:100 μm. The sharpest formate peak on the cathodic scan is obtained for a sweep potential range of 1.2 V to −1.0 V.


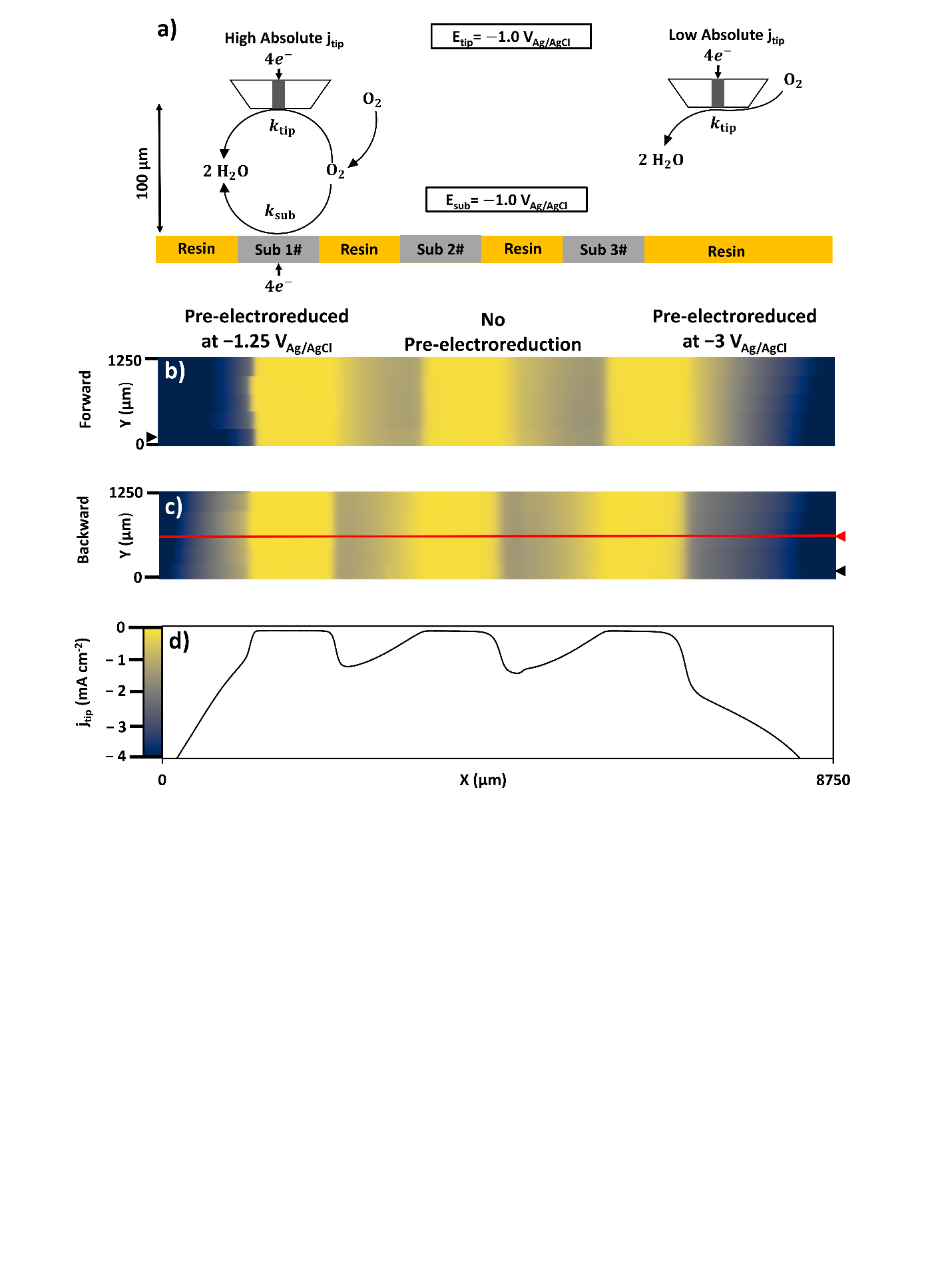


Supplementary Figure 8: Redox competition (RC) SECM scan over Sn/SnO_x_ array in air satd. 0.1 M KCl solution. a) Diagram of tip substrate RC reaction system, b) Forward scan, c) Backward scan, d) Single line scan. Tip scan rate: 100 μm s^−1^. Tip-substrate distance: 100 μm. The black arrow indicates the starting position of the tip scan. The red line in c) indicates the location of the single line scan in d). Tip current density is a uniform plateau over each substrate, indicating that tilt, substrate potential and tip-substrate distance are constant. If this was not the case, tip current density would be noticeably and consistently different from substrate to substrate over the entire scan.

**
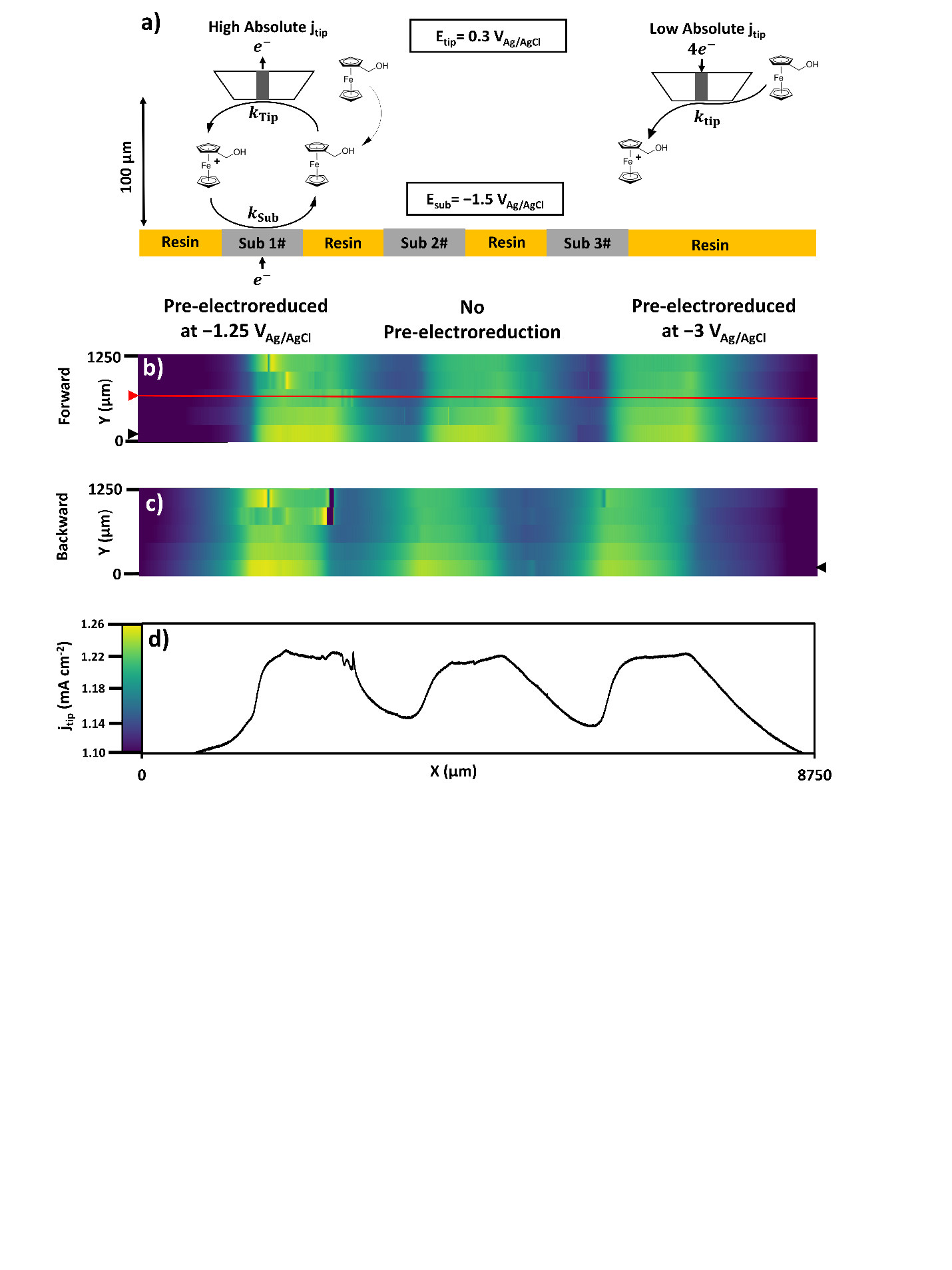
**

Supplementary Figure 9: SECM redox mediator feedback scan in 0.96 mM ferrocene methanol, 0.1 M KCl solution over Sn/SnO_x_ catalyst array. a) Diagram of tip substrate feedback reaction system b) Forward scan c) Backward scan d) Single line scan. Tip scan rate: 100 μm s^−1^. Tip substrate distance: 100 μm. The black arrow indicates the starting position of the tip scan. The red line in b) indicates the location of the single line scan in d). Tip current density is relatively constant over each substrate, indicating that tilt, substrate potential and tip-substrate distance are constant.

**Reference**

1. Sun, S. G., Clavilier, J. & Bewick, A. The mechanism of electrocatalytic oxidation of formic acid on Pt (100) and Pt (111) in sulphuric acid solution. *J. Electroanal. Chem. Interfacial Electrochem.* **240**, 147–159 (1988).

2. John, J., Wang, H., Rus, E. D. & Abruña, H. D. Mechanistic Studies of Formate Oxidation on Platinum in Alkaline Medium. *J. Phys. Chem. C* **116**, 5810–5820 (2012).

3. Spendelow, J. S., Goodpaster, J. D., Kenis, P. J. A. & Wieckowski, A. Mechanism of CO Oxidation on Pt(111) in Alkaline Media. *J. Phys. Chem. B* **110**, 9545–9555 (2006).

4. Zheng, J., Sheng, W., Zhuang, Z., Xu, B. & Yan, Y. Universal dependence of hydrogen oxidation and evolution reaction activity of platinum-group metals on pH and hydrogen binding energy. *Sci. Adv.* **2**, 1–9 (2016).

5. Durst, J. *et al.* New insights into the electrochemical hydrogen oxidation and evolution reaction mechanism. *Energy Environ. Sci.* **7**, 2255–2260 (2014).
